# Web-review: Interventional radiology

**Published:** 2008-02

**Authors:** Surg Cdr IK Indrajit

**Affiliations:** Department of Radiodiagnosis and Imaging, Army Hospital (Research and Referral), Delhi Cantt - 110 010, India

A few useful websites dealing with interventional radiology and related topics are reviewed below:

1. **Society of Cardiovascular and Interventional Radiology (SCVIR)** at http://www.scvir.org/ is a website for physicians who specialize in interventional or minimally invasive procedures. The site has numerous engrossing sections, including the one titled ‘Clinical Practice Guidelines’. These guidelines, accessible at http://www.scvir.org/clinical/index.htm#9 or alternatively at http://www.practiceguidelines.org, facilitate practice principles producing high quality interventional radiology care. This online educational compendium ‘attempts to define practice principles that generally should assist in producing high quality medical care.’ It contains quality improvement documents, consensus documents, policy and position statements, technology assessment documents, and standards from outside organizations.
2. The **Indian Society of Vascular and Interventional Radiology (ISVIR)** formed at Trivandrum in November 1997 is ‘a platform for exchange of information regarding catheter-based radiological procedures.’ Available at http://www.isvir.in/, the web portal has sections titled ‘About ISVIR,’ ‘Events and Meetings,’ ‘Information for Patients,’ and a registry. The registry, titled ‘National registry for vascular interventional radiological procedures in India,’ is available at http://161.58.194.156/isvreg2/ and efforts are underway to commence disease and technique-specific data collection.
3. The **Indian Society of Neuroradiology** at http://isnrindia.org/pages/index.php was ‘established to provide a platform for the exchange of knowledge in a rapidly advancing area of medical science.’ The web portal has numerous sections like ‘Profile,’ ‘Activities,’ ‘Programs,’ ‘News,’ ‘Knowledge Base,’ and ‘Careers.’ The section on ‘Knowledge Base’ currently has material on the history and growth of neuroradiology in the last decade, neuroradiology and imaging at AIIMS (New Delhi), Lucknow, and Shree Chitra Tirunal Institute for Medical Sciences & Technology.
4. **Endovascular.org** at http://www.endovascular.org/ is an interesting site which offers content and relevant information about circulatory diseases and vascular interventions. An initial registration is mandatory. ‘EndoChat Room,’ ‘Experts Online,’ an interactive ‘Ask the Experts’ section, and ‘Interactive Challenge’ are a set of interesting sections.
5. The **Journal of Endovascular Therapy** is accessible at http://www.jevt.org/. Formerly called Journal of Endovascular Surgery, this online journal has free access to full-text articles. A useful index of archives is also available.
6. **Intravascular Imaging** is accessible at http://www.remedica.com/ii/us/ii/index.html. Offered free from Remedica Publishing, Ltd., a mandatory registration is required. ‘This journal, which is read by more than 10,000 clinicians worldwide, is designed as a guide to the now extensive but often dispersed literature on vascular investigations available to interventional clinicians.’ The editorial team reviews significant articles on intravascular imaging topics. Similarly, ‘Interventionalist and Interventional News’ at http://www.interventionalist.org is a product of landmark collaboration between the SCVIR (North America) and CIRSE (Europe) and was ‘created to enhance interdisciplinary communication, to deliver up-to-date articles on new developments, and provide news and information to the interventional community around the world.’
7. **Vascular Disease Foundation** at http://www.vdf.org/ deals with the all-important condition of peripheral arterial disease which clearly is ‘underdiagnosed and undertreated.’ The site has numerous features, such as information on peripheral arterial disease and smoking. **Legs for Life** at http://www.legsforlife.org ‘is a national screening program for peripheral vascular disease (PVD), dedicated to improving the cardiovascular health of the community. Legs for Life is supported by the American Radiological Nurses Association (ARNA), the Society for Vascular Nursing (SVN), and the Cardiovascular Radiology Council of the American Heart Association.’ Additionally, the site has facts about abdominal aortic aneurysms and a section titled ‘Know your risk for PVD.’
8. **Cathlab** at http://www.cathlab.com/ is an engrossing website that focuses on interventionists by combining an enormous database, interactive media, and the World Wide Web. The site has sections on education organizations, publications, etc. The section on companies at http://www.cathlab.com/companies/ is a handy and useful database comprising the makers of angioplasty balloons, catheters, contrast media, guide wires, stents, etc.
9. **PTCA Angioplasty** at http://www.ptca.org is an interactive site that features ‘News’ (late-breaking news available online), ‘Forum’ (a discussion area on a wide range of topics), and ‘Interview’ (interviews with pioneers in interventional cardiology). The video section is ‘an visual archive of clips, containing exclusive interview material with Andreas Gruentzig, dramatic narratives about the first angioplasty, and the ‘accidental’ invention of coronary angiography; all clips are available in the Real Video format for instant ‘streaming.’ An exceptionally good set of links are at hand at http://www.ptca.org/links.html.
10. **Interventional Neuroradiology Service** at Wake Forest University School of Medicine is highlighted at http://www.rad.bgsm.edu/interventionneurorad/Intervent_neurorad.htm. **Endovascular Stenting for Carotid Bifurcation Stenosis** is a feature on carotid angioplasty and stenting by Cheung *et al*. from the Sunnybrook Health Science Centre, University of Toronto, Canada and is available at http://www.sunnybrook.utoronto.ca/~medimg/carotid_stent/stent2.html.

## End piece

The **Dotter Interventional Institute** at http://www.ohsu.edu/dotter/ offers educational sections like ‘New Interventional Technology,’ ‘Fibroid Embolization,’ ‘Endoluminal Treatment of AAA,’ ‘Carotid Stenting,’ and ‘TIPS.’ **Interventional Musculoskeletal Procedures** is accessible at http://www-ulpmed.u-strasbg.fr/irws/index.html. Gangi *et al*. from the Strasbourg Université at France has authored this multimedia tutorial.

There are numerous online teaching files in interventional radiology. Among them, a few salient sites include **Interventional Radiology Teaching File** at http://rpiwww.mdacc.tmc.edu/di/irtf.html., **Interventional rounds** at http://www.mirs.org/interv.htm., **Radquiz Vascular Radiology Cases** at http://www.radquiz.com/vascular.Teaching.htm., **Angiography and Interventional Modules** at http://www.t2star.com/angio.html., and **UNC Cardiovascular Radiology Teaching File** at http://sunsite.unc.edu/jksmith/UNC-Radiology-Webserver/CVInterventional.html

**MyPACS** at http://www.mypacs.net/repos/mpv3_repo/static/m/Home/ is ‘a free service to the international community funded, in part, by the National Institutes of Health.’ It is essentially a web storehouse of radiology cases with images, comprising contributions from radiologists from 2500 institutions in 75 countries, at this point in time. Registration with a free account and a web browser allows viewing and/or sharing of radiology cases. Supported by Vivalog Technologies on a Linux platform, the site with a user-friendly interface promotes sharing of ‘knowledge through the use of content management technology.’

**Aunt Minnie** at http://www.auntminnie.com/ is ‘the largest and most comprehensive community website’ for professionals worldwide in the medical imaging industry. Launched in November 1999 by Dr. Phillip Berman, the website provides a forum for continuous exchange of the latest medical imaging news and information. It showcases the dynamic changes that radiology is witnessing, day in and day out. The Web portal has many diverse and useful sections like ‘Buyers Guide,’ ‘Radcasts,’ ‘Conferences,’ ‘Communities,’ ‘Regulatory Updates,’ and so on. Sections focusing on radiology education include ‘Rad Path,’ ‘Teaching Files,’ ‘Boards Review Questions,’ ‘Case of the Day,’ ‘Practice Guidelines,’ and ‘White Papers.’ However, for sheer convenience, two handy Web pages that serve as useful starting points are the **Site Map** available at http://www.auntminnie.com/help/sitemap.asp and an **Archive Map** available at http://www.auntminnie.com/Articles/archivemap.htm. Incidentally, ‘Aunt Minnie’ is a term coined by the legendary Dr. Ben Felson, University of Cincinnati, in the 1940s, and is customarily used to describe ‘a case with radiologic findings so specific and compelling that no realistic differential diagnosis exists.’

